# Dental implants and risk of bleeding in patients on oral anticoagulants: a systematic review and meta-analysis

**DOI:** 10.1186/s40729-021-00364-5

**Published:** 2021-08-25

**Authors:** Basim E. S. Dawoud, Samuel Kent, Oliver Tabbenor, Pynadath George, Jagtar Dhanda

**Affiliations:** 1 Department of Oral & Maxillofacial Surgery, North Manchester General Hospital, Manchester Foundation Trust, Manchester, UK; 2 Department of Oral and Maxillofacial Surgery, Aberdeen Royal Infirmary, Aberdeen, UK; 3 Department of Oral and Maxillofacial Surgery, Manchester Foundation Trust, Manchester, UK; 4 Department of Oral Surgery, Edinburgh Dental Institute, Edinburgh, UK; 5 Department of Oral and Maxillofacial Surgery, Queen Victoria Hospital NHS Foundation Trust, East Grinstead, UK

**Keywords:** MeSH terms, Dental implant, Anticoagulation, Haemorrhage

## Abstract

**Background:**

Dental implant placement is safe and predictable, yet optimal management of anticoagulated patients remains controversial. Whilst cessation of anticoagulation pre-operatively should decrease risks of bleeding, risk of thrombosis increases. We aim to define risk of bleeding in patients on oral anticoagulation who are undergoing dental implant placement, in order to establish best management.

**Methods:**

This systematic review is registered with the National Institute for Health Research (NIHR) PROSPERO database (Registration No: CRD42021233929). We performed a systematic review as per Preferred Reporting Items for Systematic Reviews and Meta-Analyses (PRISMA) guidance. Studies were identified using an agreed search strategy within the OVID Gateway (this included Pubmed, MEDLINE, Cochrane Collaborative). Studies assessing bleeding complications in patients who were undergoing dental implant placement were selected. The primary outcome was bleeding events in anticoagulated patients undergoing dental implant placement. Secondary outcomes included any complication requiring further intervention.

**Results:**

We identified 182 studies through screening, and after review of titles and abstracts reduced this to 8 studies. In these studies, 1467 participants received at least 2366 implants. Studies were analysed for quality using the ROBINS-I risk of bias tool. Four studies were retrospective case reviews, and four were prospective reviews, three of which also blinded the operator to anticoagulation status. There was significant heterogeneity between the included studies. Meta-analysis showed an increased risk of bleeding (RR, 2.30; 95% CI, 1.25-4.24 p = 0.37 I = 7%) when implants were placed in the presence of anticoagulation however these were not clinically significant haemorrhagic events.

**Conclusion:**

The continuation of anticoagulants peri-operatively during dental implant surgery does increase the risk of clinically non-significant peri- and post-operative bleeding. Dental implant surgery encompasses a broad spectrum of procedures ranging from minor to more invasive surgery with simple local haemostatic measures mitigating the risk of bleeding. The decision to discontinue anticoagulants prior to dental implant surgery must consider patient and surgical factors with the clinician undertaking a risk-balance assessment.

## Introduction

Dental implant placement is safe and predictable, yet optimal management of anticoagulated patients remains controversial. Weighing up the risks and benefits of placing dental implants in anticoagulated patients and whether stopping or pausing anticoagulation peri-procedurally mitigates bleeding risk remains unclear. Regular review of all available evidence, with meta-analysis, allows better understanding of these risks and benefits.

The volume of dental implant placement has proliferated in the past 20 years, with over 10,000 mandibular implants placed per year in the UK [1]. Refinements in implant design and procedural protocols have decreased complication rates to around 2%, yielding 1- and 5-year survival rates of around 99% and 94% respectively [2]. However, variation in practice remains, particularly regarding medical management of patients undergoing implant placement. Detailed review of the evidence may allow further gains in optimising management.

Management of the anticoagulated patient has evolved with the introduction of direct oral anticoagulants (DOACs) and concurrent reduction in use of warfarin. DOACs have a number of advantages over warfarin—they have been shown to cause fewer life threatening haemorrhages than warfarin [3], have shorter half-life, do not require alterations in daily dosing, and reversal agents are now available for all DOACs [4]. Yet, there are not yet clear protocols for management of patients on DOACs undergoing surgical procedures. Whilst cessation of anticoagulation pre-operatively would decrease bleeding risk, it would also increase risk of embolic events [5].

Equipoise exists in the literature, with some authors advocating no alteration to anticoagulation protocols when placing dental implants [6], and others advising that pausing anticoagulation is necessary [7]. Systematic review of the literature and meta-analysis is required to define bleeding risk of anticoagulated patients undergoing dental implant placement, and hence determine optimal management of this patient group.

## Methods

This systematic review is registered with the National Institute for Health Research (NIHR) PROSPERO database (Registration No: CRD42021233929) and has been designed and reported in accordance with the preferred reporting items for systematic reviews and meta-analyses (PRISMA) [8].

### Search strategy

Searches were conducted via Ovid Gateway (including MEDLINE, PubMed and Cochrane Collaboration), PubMed from inception to January 2021, ISI Web of Science, from inception to June 2020. Search terms included “dental implants”, “zygomatic implants”, “oral implants” and “anticoagulants”, “warfarin”, “direct oral anticoagulants”, “DOAC”, “rivaroxaban”, “apixaban”, “dabigatran” and “bleeding”, “complications”.

### Study selection

Abstract screening was undertaken by two authors (BD/SK) to ascertain relevance to the research question. The full texts were obtained and then screened independently.

### Data extraction

Reviewing authors collected data regarding study type, number of participants and implants in the anticoagulated group and none-anticoagulated (control) group respectively and peri-operative anticoagulation protocol. Further data included reported outcome complications (haemorrhage, haematoma formation, purpura, bleeding requiring further intervention). Corresponding authors were contacted where data was missing, or articles were inaccessible.

### Outcomes

The primary outcome was any significant bleeding (as defined and reported by each respective study) requiring treatment. This may have been reported within the immediate peri-operative phase or bleeding complications within 7 days of primary surgery.

### Risk of bias assessment

Studies were assessed for risk depending upon their methodology. For non-randomised studies the The Risk Of Bias In Non-randomised Studies of Interventions (ROBINS-I) tool was used [9]. This was stratified into four escalating categories with 0 and 1 representing no information and low risk respectively to 4 indicating serious risk of bias.

### Data analysis

Analysis was carried out via direct comparison meta-analysis using Review Manager ® version 5 [10]. Analysis was performed to calculate the risk ratios (RR) of any reported bleeding complication with a confidence interval (CI) of 95%. Clinical and statistical heterogeneity was assessed using a random-effects model and I^2^ respectively, where an I^2^ score of 0 indicates complete homogeneity between studies. Studies and data were split into two subgroups following reported peri-operative anticoagulation protocol. These were split into studies which continued and discontinued anticoagulant therapy peri-operatively respectively.

## Results

### Study selection

**Fig. 1:**
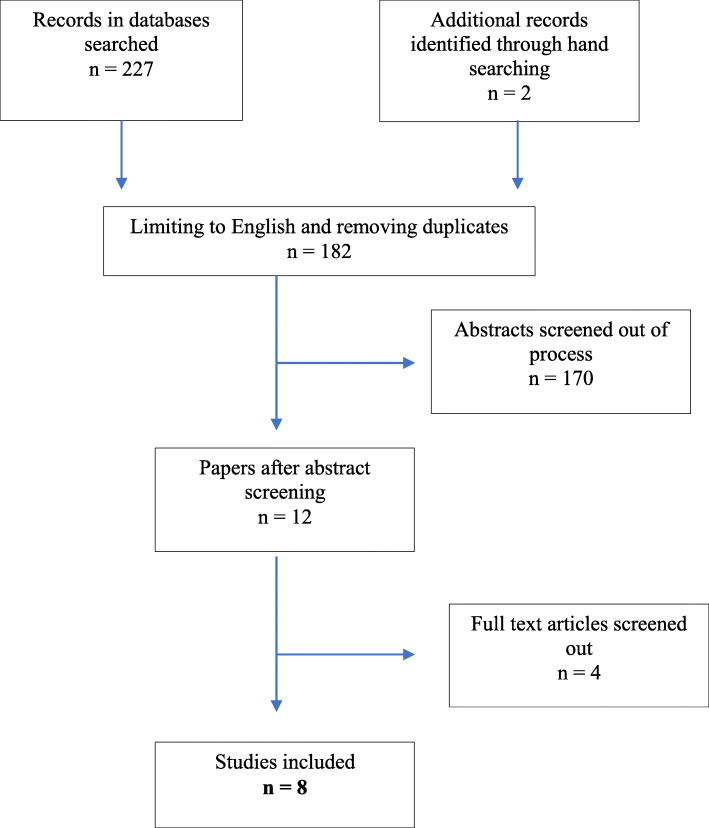
PRISMA flowchart

A total of 182 original studies following limiting to English language and removing non-duplicates were identified from the searches and from screening. Abstract screening removed 170 studies not relevant to the study. Full texts of 12 articles were obtained for further assessment, resulting in exclusion of a further 4 studies. Finally, 8 studies [6, 11–17] met eligibility criteria for this review and were included for full analysis (Fig. 1).

### Study characteristics

All articles included were observational cohort studies. There were 4 retrospective [6, 11, 12, 17] and 4 prospective [13–16]; of the prospective studies, 3 [13, 14, 16] blinded the operator from which patients were taking anticoagulants. A total of 1467 participants were included with a minimum of 2366 implants placed across all studies. There were 3 studies [6, 15, 17] which did not report the number of implants placed within their respective cohorts; therefore, the total number of implants was under-estimated to reflect a singular implant for the number of participants within those studies. Further information on the study characteristics are included in Table 1.

**Table 1:** Included studies characteristics

Study	Study type	Participants (n)	Evidence level [18]	Maximum implants placed per participant (n)	Implant brand/type placed	Mucoperiosteal flap raised or flapless surgery	Immediate or delayed loading	Bleeding events reported (total number anticoagulated) ^c^	Bone graft (n)
**Manor 2020** [6]	Retrospective observational cohort study	193	2b	Not reported	Not reported	Mucoperiosteal flap	Delayed	4 ^d^ (72)	27
**Sannino 2020** [12]	Case-control study	120	3b	4 (full arch)	CSR-DAT, Sweden & Martina, Due Carrare, Italy	Mucoperiosteal flap posteriorly only	Immediate	Control: 1^e^ (40) Case: 11^a^ (40) Case: 3^b^ (40)	None reported
**Galletti 2020** [11]	Retrospective observational cohort study	12	2b	4-6 (full arch)	Ossean®; Intra-Lock International®, Inc., Boca Raton, FL, USA	Mucoperiosteal flap	Immediate	3^b^ (12)	Not specified how many grafted
**Okamoto 2018** [17]	Retrospective observational cohort study	289	2b	1-6	Not reported	Not reported	Not Reported	4^a^(19)	Patients excluded if grafted
**Gomez-Moreno 2018** [14]	Case-control study	71	3b	Number of implants per patient not specified	Straumann® AG, 8, 10 or 12 mm in length and 3.3 or 4.1 mm in diameter.	Mucoperiosteal flap	Delayed	Control: 2 (42) Case : 2^f^ (29)	Control: 9 Case: none reported
**Clemm 2016** [15]	Case-control study	564	3b	Number of implants per patient not specified	Not reported	Mucoperiosteal flap	Delayed	Control: 3 (447) Case: 4^d^ (117)	Control: 77 Case : 16
**Gomez-Moreno 2016** [13]	Case-control study	57	3b	1-3	Straumann® AG, 8, 10 or 12 mm in length and 3.3 or 4.1 mm in diameter.	Mucoperiosteal flap	Delayed	Control: 2^d^ (39) Case: 1^b^ (18)	Control: 9 Case: none reported
**Bacci 2011** [16]	Case-control study	161	3b	Number of implants per patient not specified	Not reported	Mucoperiosteal flap	Delayed	Control: 3 (109) Case: 2^a^ (52)	Control: 66 (implants) Case: 30 (implants)

^a^Warfarin | ^b^Rivaroxaban | ^c^Moderate events | ^d^ Combination of DOACs/warfarin or antiplatelets | ^e^No anticoagulant | ^f^Dabigatran

In order to minimise clinical heterogeneity with study protocols, the analysis was split into two subgroups. The studies were split dependant upon their peri-operative protocols in which one group (1) continued anticoagulation peri-operatively without a pause [6, 12, 13, 15–17] and those which (2) discontinued in the immediate pre-operative period [11, 14] and restarted 6-12 h post-operatively.

There was variability in the oral anticoagulants included within the studies. In total, three studies [11, 13, 14] compared a single direct oral anticoagulant (DOAC) with a control group of no anticoagulant with three studies [6, 12, 15] grouping patients on either warfarin or any other DOACs. One [16] compared warfarin alone with a control group of patients not taking warfarin and one [17] study comparing patients on warfarin and an antiplatelet with a control group of no anticoagulants or antiplatelets.

There was variability when reporting outcome measures (complications) between the studies.

One study [6] required patients to self-report bleeding and of those who required urgent clinical assessment, the bleeding was quantified subjectively. Four studies [11, 13, 14, 16] analysed bleeding in accordance to criteria set out by Bacci et al. [16] which categorises bleeding complications into four stages with escalating levels of haemostatic control. One [12] study qualified bleeding within 24 h as a primary outcome measure with anything > 24 h considered a secondary outcome with associated features of haematoma, purpura or ecchymoses. One study [17] did not qualify the criteria for bleeding complications and was based on subjective assessment of the patient. One study [15] defined their own criteria of bleeding as low, moderate or severe with escalating levels of intervention for haemostatic control. All included studies reviewed patients at least once within a 7-day post-operative period or same day assessment in the case of urgent bleeding.

### Risk of bias

**Fig. 2:**
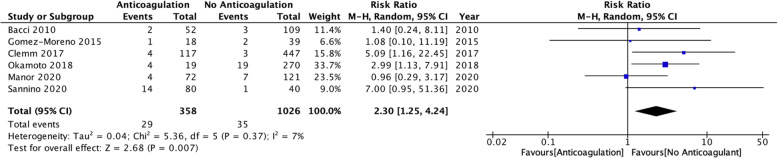
Forest plot comparing continuation of anticoagulation therapy peri-operatively with controls of no anticoagulation. Mean relative risk and pooled relative risk is represented by the blue squares and black diamond respectively

The risks of bias within the methodology of the included studies are shown in Fig. 2 and Table 2. RCTs are assessed in Fig. 2 in accordance with Cochrane Collaboration’s tool for assessing bias [19] and non-randomised studies analysed using the Risk of Bias in Non-Randomised Studies – of Intervention tool (ROBINS-I) [9] (Table 2).

**Table 2:** ROBINS-I tool risk of bias in non-randomised studies

Study	Confounding	Selection of participants	Intervention classification	Deviation from intervention	Missing data	Measurement of outcome	Selection of reported result	Overall
Manor 2020 [6]	2	2	3	2	1	3	2	2
Sannino 2020 [12]	2	2	1	1	1	3	2	1
Galletti 2020 [11]	3	3	2	2	3	2	2	3
Okamoto 2018 [17]	3	3	2	2	2	3	2	3
Gomez-Moreno 2018 [14]	2	2	2	1	1	2	2	2
Clemm 2016 [15]	2	1	2	2	1	2	1	1
Gomez-Moreno 2016 [13]	2	3	1	1	1	3	3	2
Bacci 2011 [16]	2	3	1	1	3	2	1	2

Risk of bias assessment: 0 – No information | 1 – Low | 2 – Moderate | 3 – Serious | 4 – Critical

### Effects of intervention

**Fig. 3:**
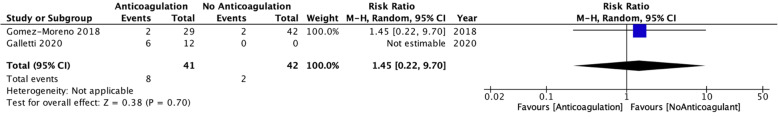
Forest plot comparing pausing of anticoagulation therapy peri-operatively with controls of no anticoagulation. Mean relative risk and pooled relative risk is represented by the blue squares and black diamond respectively

Table 3 summarises the findings between the respective peri-operative protocols utilised for management of anticoagulation in dental implant surgery. When comparing continuation of anticoagulants peri-operatively to the control of no anticoagulation (Fig. 2), the relative risk of the studies demonstrate an increased risk of peri-operative bleeding complications when anticoagulants are continued (RR, 2.30; 95% CI, 1.25—4.24 p = 0.37 I = 7%); however, the quality of evidence is low with risk of bias throughout (Table 2). Despite the increased risk of bleeding, across the entire cohort of 1467 participants, only two participants required hospitalisation for the severity of bleeding [15] of which one participant was anticoagulated and the other was not. All the studies that reported bleeding complications were managed with local haemostatic measures without the need for further interventions. There was insufficient data to perform a meaningful meta-analysis on whether pausing anticoagulation (Fig. 3) reduces the risk of bleeding complications (RR 1.45, 95% CI, 0.22-9.70).

**Table 3:** Summary of the interventions

Anticoagulant groups	Incidence of adverse events (total)		Relative risk adverse event (95% CI)	Number of participants (studies)
	Group A (%)	Group B (%)		
**Continuation of anticoagulants (group A) vs no anticoagulant (group B)**	29 (8.1)	35 (3.0)	2.30 [1.25, 4.24]	1384 (6)
**Pause of anticoagulant (group A) vs no anticoagulant (group B)**	8 (19.5)	2 (4.7)	1.45 [0.22, 9.70]	83 (2)

## Discussion

There are different approaches to the management of anticoagulation in dental implant surgery. Practice with DOACs ranges from continuing anticoagulation, pausing anticoagulation prior to the day of surgery or bridging protocols with low molecular weight heparin (LMWH). National guidance classifies dental implant surgery as higher risk of post-operative bleeding complications [7]. The Scottish Dental Clinical Effectiveness Programme (SDCEP) guidance acknowledges the paucity of high quality data to support recommendations given in the guideline. For patients on DOACs, recommendations for procedures with high risk of bleeding complications are to withhold the morning dose of the drug [7]. Furthermore, for patients on warfarin, the advice is to treat patients with international normalised ratios (INR) of ≤ 4 (checked within 24 h of surgery) without a pause in their anticoagulation. Both these recommendations are based on low quality evidence as acknowledged in the guideline.

Implant procedures range from simple immediate single placements following dental extraction to more complex bi-maxillary oral reconstructive surgery (zygomatic or pterygoid implants) with associated grafting procedures. The nature of bleeding risk is not just dependent upon anticoagulation status but also of how invasive the surgery is. Most of the studies included did not stratify groups into the respective intervention to assess bleeding risk of procedure specific interventions. This is an important factor when considering a pause of the patients’ anticoagulation.

There are no randomised controlled clinical trials evaluating the effect of anticoagulation on bleeding; however, there are prospective blinded studies [13, 14, 16]. These studies continued anticoagulation peri-operatively and blinded only the surgeon to group allocation (anticoagulated and none anticoagulated). None of the included studies performed power calculations or intention to treat analysis.

The evaluation of bleeding within the peri-operative period of the included studies is fraught with bias. Bacci et al. [16] defined a criteria in which to objectively assess bleeding with an escalating level of intervention to reflect the severity of bleeding. This scale is a four tier system graded from no bleeding, slight bleeding (defined as a slight ooze) managed with compressive gauze only. The moderate category is defined as bleeding with large clots disrupting the surgical field requiring additional haemostatic measures, and severe are categorised as patients requiring systemic medical management to achieve haemostasis [16].

Some of the included studies [11, 13, 14, 16] adopted the grading published by Bacci et al. [16] as a more objective analysis and subsequently reported bleeding complications in accordance with this scale. The variability in the means of reporting bleeding complications creates heterogeneity in what is reported in the literature and may lead to an over or under reporting of complications.

Other meta-analyses have demonstrated conflicting relative risk ratios in patients on oral anticoagulants undergoing dental surgery. Shi et al. [20] reported an increase risk of bleeding in patients on oral anticoagulants compared to a control group of no anticoagulant, whilst a meta-analysis by Nematullah et al. [21] reported no significantly increased risk of bleeding in patients who continue warfarin therapy to those who discontinue. Furthermore, other research has been conducted to assess the risk of bleeding for oral surgical procedures in patients on oral anticoagulants [22–24]; however, there remains a degree of uncertainty due to discrepancies in methodology and outcome measures reported. These studies did not exclusively assess the risk of bleeding in patients undergoing dental implant surgery but rather a multitude of different oral surgical procedures.

Clearly, the decision to discontinue anticoagulation for dental implant surgery must not be taken lightly. There is evidence that discontinuation of anticoagulant therapy may increase the risk of venous thrombo-embolism causing significant morbidity or even mortality for patients [5, 25]. A study by Wahl et al. of 5431 patients undergoing dental surgical procedures, 2763 had warfarin reduced or withdrawn peri-operatively and there were subsequently 22 (0.8%) thrombo-embolic and 6 (0.2%) fatal complications [5]. The indication for anticoagulation could be for either prophylactic or therapeutic purposes and therefore must be considered carefully in the surgical consultation. Discontinuation of anticoagulation must be balanced with individual bleeding risk and patients considered on an individual basis.

All the included studies reinforce the importance of local haemostatic measures when managing patients on oral anticoagulants. Throughout the entire cohort included in this study, only two patients (one within the anticoagulated and other in the non-anticoagulated group respectively) [15] required hospitalisation due to the severity of haemorrhage, with all other studies reporting managing bleeding with simple local measures. This reinforces national guidelines which advise the prophylactic use of local haemostatic measures following oral surgical procedures [7].

### Limitations

Several of the included studies had systematic differences leading to variability in reporting of outcomes. This meta-analysis includes patients undergoing all forms of dental implant surgery; however, this is a broad range from single to multiple placements. Only one study [12] considered full arch rehabilitation as the modality of surgery when comparing anticoagulated patients with the remainder of the studies containing a mix of single and multi-implant placements. Single implant placements are considered far less invasive than a full arch osseo-integrated rehabilitation or zygomatic implants. None of the studies included zygomatic or pterygoid implants and our findings should not be extrapolated to encompass this patient group. Future clinical studies show aim to ascertain the risk of bleeding complications from higher risk and more invasive dental implant surgery.

This systematic review only considered single anticoagulant vs no anticoagulation. There is evidence that dual antiplatelets can exacerbate bleeding leading to a higher risk of complications [26, 27]. Furthermore, this review did not take into consideration patients who take anticoagulants in combination with antiplatelets. There is evidence that a combination of these two drugs can increase bleeding risk [28]; however, this has not been shown in studies for patients undergoing oral surgical procedures.

## Conclusions

Based on the available data and within the limitations of the published studies included in this meta-analysis, the continuation of anticoagulants peri-operatively during dental implant surgery does increase the risk of clinically non-significant peri- and post-operative bleeding. The decision to discontinue anticoagulants prior to dental implant surgery must be a patient specific one and involve a careful risk balance assessment. This should be done in consultation with the patient and where necessary their prescribing physician. All the studies demonstrate that any bleeding complications following dental implant surgery can easily be managed with local haemostatic measures. Dental implant surgery ranges from smaller interventions which carry a lower risk of bleeding to more invasive full mouth surgical rehabilitation such as bimaxillary and zygomatic implants. Further research should aim to reflect the respective bleeding risks for anticoagulated patients for these more invasive surgical procedures than that of single implants. This will help the development of  more tailored guidance specific to procedures that may potentially carry a higher risk of bleeding.

## Data Availability

The datasets of the current study are available from the corresponding author on reasonable request.

## Abbreviations

UK: United Kingdom
DOAC: Direct oral anticoagulants
NIHR: National Institute for Health Research
PRISMA: Preferred Reporting Items for Systematic Reviews and Meta-analyses
ROBINS-I: The Risk Of Bias In Non-randomised Studies of Interventions
LMWH: Low molecular weight heparin
INR: International normalised ratio
RR: Risk ratios
CI: Confidence intervals
RCT: Randomised controlled trial
SDCEP: Scottish Dental Clinical Effectiveness Programme
PROSPERO: International Prospective Register of Systematic Reviews
